# Clinical-histopathological and molecular study of ovine pulmonary adenocarcinoma in Awassi sheep in Al-Qadisiyah Province, Iraq

**DOI:** 10.14202/vetworld.2019.454-458

**Published:** 2019-03-26

**Authors:** Khalefa Ali Mansour, Saad Hashim Al-Husseiny, Qassim Haleem Kshash, Asaad Jassim

**Affiliations:** Department of Veterinary Internal Medicine, College of Veterinary Medicine, University of Al-Qadisiyah, Iraq

**Keywords:** Jaagsiekte sheep retrovirus, ovine pulmonary adenocarcinoma, polymerase chain reaction

## Abstract

**Aim:**

This study aimed to conduct a clinical-histopathological and molecular evaluation of ovine pulmonary adenocarcinoma (OPA) in Awassi sheep in various regions of Al-Qadisiyah Province, Iraq.

**Materials and Methods:**

A total of 150 sheep were clinically evaluated, and the wheelbarrow test was performed. 100 samples (35 blood, 25 lung tissue, 20 lymph node, and 20 lung fluid samples) were randomly selected from living and slaughtered sheep. All samples were subjected to polymerase chain reaction (PCR). Histopathological examinations were performed for four lung tissue and two lymph node samples.

**Results:**

A diagnosis of OPA was made based on the results of the clinical examination and the clinical signs shown by the animals, such as dyspnea, polypnea, coughing, mucous nasal discharge, moist rales on auscultation of the affected lungs, and emaciation. Interestingly, the animals tested positive for the wheelbarrow test, with frothy nares accompanied by profuse and clear lung fluid. Histopathological examination showed various lesions such as glandular transformation in the lung tissues and emphysema. Moreover, lymph nodes showed marked follicular atrophy and necrosis-associated lymphocyte infiltration in the affected tissues. PCR revealed that 25% of the samples including eight (22.8%) blood, five (20%) lung tissue, five (25%) lymph node, and seven (35%) lung fluid samples were positive for Jaagsiekte sheep retrovirus; this result was highly significant.

**Conclusion:**

The results of our study indicated that in Iraq, OPA diagnosis should be based on pathological findings and results of advanced procedures such as PCR.

## Introduction

Ovine pulmonary adenocarcinoma (OPA) is a contagious disease of adult sheep caused by infection with Jaagsiekte sheep retrovirus (JSRV). The disease clinically manifests as tumor lesions in the lung. It is associated with a high rate of infection and deaths in the affected sheep, resulting in severely compromised health in the affected animals and subsequently massive losses to the economy of the areas with the infection [1,2]. Livestock from only a few countries, i.e., Iceland, the Falkland Islands, New Zealand, and Australia, have not had this disease in recent times. However, there was an extreme outbreak in Iceland, in 1930, with an infection rate of ~30% in sheep [3]. JSRV is a tumorigenic retrovirus that affects the epithelial cells of the lung; infection with JSRV is characterized by the presence of a high number of cancer masses in the affected lungs [4,5].

The disease affects mostly lambs but can develop in adults as well; affected adults show respiratory signs including nasal discharge, dyspnea, polypnea, coughing, and profuse fluid accumulation in the lungs [6,7]. The disease requires some time to transition from subclinical to clinical levels in a sheep flock as the virus induces a state of immunotolerance as it embeds its DNA into the host DNA [8]. Thus, serological screening tests during the subclinical period may provide false-negative results and subsequently result in severe disease that could cause increased livestock deaths and economic losses.

To the best of our knowledge, thus far, only two studies on JSRV-induced OPA have been performed in Iraq: First, in Mosul province using histopathological analyses [9] and the second in Al-Qadisiyah Province using polymerase chain reaction (PCR) [10]. Thus, this study aimed to confirm the presence of this disease in sheep in Al-Qadisiyah Province using PCR in addition to clinical and histopathological evaluations.

## Materials and Methods

### Ethical approval

The ethics review board of Al-Qadisiyah, college of veterinary medicine approved this study design.

### Clinical examination

Several herds in different rural areas of Al-Qadisiyah Province were clinically examined. Case history and clinical signs were recorded, such as dyspnea, polypnea, coughing, mucosal nasal discharge, moist rales on auscultation, and emaciation. The animals tested positive in the wheelbarrow test, with frothy nares accompanied by profuse and clear lung fluids.

### Sample collection

A total of 150 animals were clinically inspected during 2016-2017 for the presence of clinical signs related to OPA. 100 samples (35 blood, 25 lung tissues, 20 lymph nodes, and 20 lung fluids) were obtained from living and slaughtered sheep.

### Histopathological examination

Six tissue samples (four lung and two mediastinal lymph node samples) measuring approximately 1 cm^3^ in size each were collected from infected sheep and fixed in 10% neutral buffered formalin according to previously described procedures [11].

### Viral RNA extraction and PCR

Viral RNA was extracted from the samples using AccuZol^TM^ RNA extraction kit (Bioneer, Korea). PCR was performed using primers specific for the envelope protein-coding gene, KT279065.1. The primers (Bioneer Company, Korea) targeted a 382-bp region in the gene: F, CCGGAAAGAGATCGTACCGT and R, TAAGGAACACAAGCTCGGGG. The AccuPower^®^ RT-PCR PreMix (Bioneer, Korea) was used with the extracted RNA to obtain the reaction solutions. PCR was performed according to the manufacturer’s instructions. The amplification of the required region of the gene was performed in one tube with the reverse transcription process using the following thermocycler conditions: 50°C for 15 min, 95°C for 5 min, 95°C for 20 s, 58°C for 30 s, 72°C for 1 min, and 72°C for 5 min for one cycle of primary denaturation; 30 cycles of main denaturation, annealing, and main extension; and one cycle of final extension. Electrophoresis was performed using 2% agarose gel stained with ethidium bromide and a 100 volt-80 Amp current. The products were visualized under an ultraviolet-based imager (Vilber Lourmat, France).

### Statistical analysis

The obtained data were statistically analyzed for significant differences using ANOVA in SPSS software (version 7).

## Results

### Clinical examination

**Figure-1:**
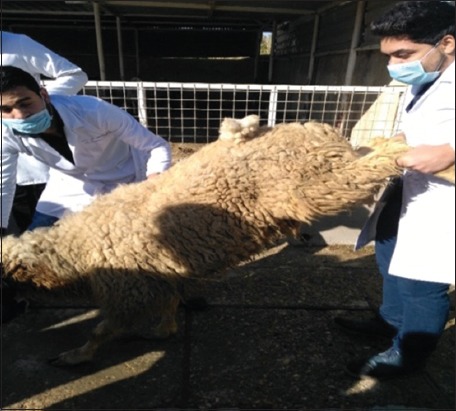
Lung fluid collection during wheelbarrow test for affected sheep with ovine pulmonary adenocarcinoma.

The clinical signs shown by the affected animals, such as dyspnea, polypnea, coughing, mucosal nasal discharge, moist rales on auscultation of the affected lungs, and emaciation, indicated the presence of OPA. Furthermore, the animals tested positive for the wheelbarrow test, with frothy nares and profuse and clear lung fluids (Figure-1).

### Gross and histopathological results

**Figure-2:**
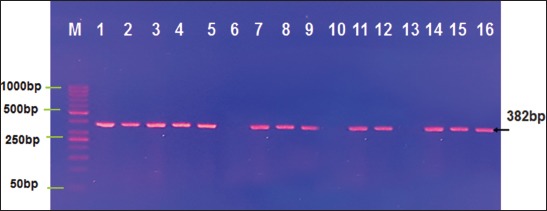
Image of 2% agarose gel electrophoresis displays Jaagsiekte sheep retrovirus. M is the ladder (50-1000 bp). Lanes (1-5, 7-9, 11, 12, and 14-16) are the positive samples at 382 bp polymerase chain reaction product.

The gross examination showed that the trachea was filled with frothy fluid, and the lungs are enlarged, heavy, and edematous. Palpation showed consolidated foci and the affected areas appeared darker than the neighboring normal tissue. In addition, mediastinal lymph nodes were often edematous and enlarged (Figure-2). The histopathological evaluation showed the following findings.

#### Lung tissues

**Figure-3:**
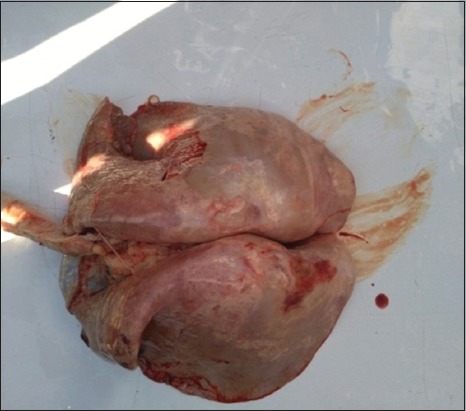
Gross appearance of ovine pulmonary adenocarcinoma lesions in lung of adult sheep showing multifocal white neoplastic nodules.

**Figure-4:**
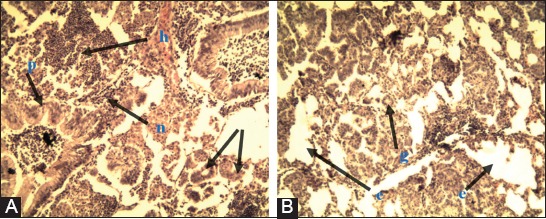
Lung in ovine pulmonary adenocarcinoma. (A) Inflammatory cell-based infiltration (n). Interstitially, neoplastic structure formation (g) and hemorrhage (h). Epithelial cell-related hyperplasia in the bronchioles (p). (B) Neoplastic structure formation (g) and emphysema (e) H and E 10×.

There was extensive inflammatory cell infiltration in the lungs. Glandular structure (neoplastic) formation and hemorrhage were observed in the interstitium. Epithelial cell hyperplasia was noted in the bronchioles. In addition, there were clear signs of emphysema (Figures-3 and 4).

#### Lymph nodes

**Figure-5:**
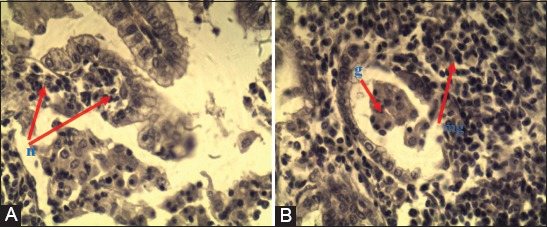
Lung in ovine pulmonary adenocarcinoma. (A) Inflammatory cell-based infiltration (n). (B) Tumor structure formation (g) and macrophage cell-based infiltration (m) H and E 40×.

Follicular atrophy, thickened fibrinous trabeculae, necrosis-associated lymphocyte infiltration, and hyperplastic proliferation were observed in the walls of blood vessels of the affected tissues (Figure-5).

### PCR results

**Figure-6:**
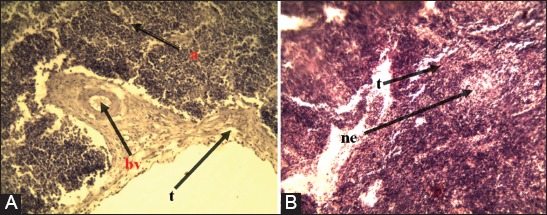
Lymph node in ovine pulmonary adenocarcinoma. (A) Follicular atrophies (a) and hyperplastic-based proliferation in the walls of blood vessels (B) thickened fibrinous trabeculae (t) and necrotic-based lymphocyte infiltration (ne) H and E 10×.

PCR revealed that eight (22.8%) blood, five (20%) lung tissue, five (25%) lymph node, and seven (35%) lung fluid samples were positive for JSRV, the causative agent of OPA (Table-1 and Figure-6).

**Table-1:** The positive PCR JSRV in different samples of sheep.

Samples	Number of examined samples	+PCR JSRV (%)
Blood	35	8 (22.8^a^)
Lung	25	5 (20^b^)
Lymph node	20	5 (25^c^)
Lung fluid	20	7 (35^d^)
Total	100	25 (25)

The different lowercase letters refer to significant variations at p≤0.05. PCR=Polymerase chain reaction, JSRV=Jaagsiekte sheep retrovirus

## Discussion

OPA is a contagious disease of adult sheep that is caused by JSRV. The disease is clinically characterized by the formation of tumor lesions in the lung. OPA is associated with high rates of infection and deaths in the affected sheep, causing huge losses in terms of the health of the livestock and subsequently to the economy in the infected areas [1,2]. It identified the presence of OPA in sheep that were examined in Al-Qadisiyah-related territories. The disease was successfully diagnosed based on the marked clinical sign of OPA, which was also noted in a previous study [12]. Most importantly, the wheelbarrow test showed profuse amounts of fluid in the lungs, which was an outstanding sign to differentiate the disease from other respiratory conditions and diseases. These signs have also been confirmed in other studies worldwide as important for diagnosing OPA; these studies are in agreement that the pathognomonic clinical diagnostic sign of OPA is the amount of fluid in the lung that passes through the nostrils of the sheep when the animal lifts its rear quarter while lowering its head [13-16].

PCR analysis of the all the samples obtained revealed the presence of OPA in the sheep in the studied regions to be 25%, indicating the reliability of PCR for early screening of the disease, even in subclinical cases [17,18]. This result is in agreement with those of the previous studies in which the virus in the sheep was detected using PCR for the 1^st^ time in Ireland [19-21]. The finding is also supported by those of many studies in which OPA was diagnosed using PCR, such as the ones conducted in Iraq (5.55%) [10], India (8%) [12], Iran (13.75%) [22], and Northwest Iran (18%) [23]. Moreover, these findings are consistent with those of epizootic sheep diseases, reported in many countries except Australia, New Zealand, Falkland Islands, and Iceland [3]. It was identified JSRV in all samples by PCR; these findings were similar to those reported previously [10,20], in terms of chronicity, carcinogenic character, and long duration of the disease, which allow the infection to spread to most tissues. However, it was found a higher infection rate in the lung fluid samples than in the other samples, consistent with the previous results [14].

In the previous study, the virus was found in higher concentrations in pulmonary fluid than in the other organs, which increased positive results for infection in lung fluid. The infection rate in the mediastinal lymph nodes was higher than that in lung tissues and blood; this finding was in agreement with that reported previously [6]. Furthermore, the findings of a study conducted in Iran [22] were consistent with these results, in that the detection rate in blood samples was higher than that associated with lung tissue samples. Interestingly, the gross findings of the infected lung were in line with the previous results [24]: The normal architecture of the lung was disturbed with enlargement of regional lymph nodes.

The histopathological analyses were useful in supporting the results of PCR in this study. The analyses revealed tumor nodules infiltrating the alveoli with excessive infiltration of macrophages around a neoplastic focus in lung tissue as well as hypertrophic changes in lymph nodes; these findings were in agreement with previously reported ones [22,25,26]. Further, in the lymph nodes, neoplastic cells occupied the subcapsular and paratrabecular sinuses or showed locally extensive growth, replacing the normal cortex [27,28]. This study provides important data for OPA diagnosis.

## Conclusion

In this study, the diagnosis of OPA in Iraq with the clinical and histopathological findings was confirmed by PCR technique as an advanced method.

## Authors’ Contributions

SHA and QHK have identified the research question and design of the study. AJ performed the survey and collected the samples from animals. KAM carried out the necessary laboratory tests. All authors read and approved the final manuscript.
